# Risk Factors for Mortality From Late-Onset Sepsis Among Preterm Very-Low-Birthweight Infants: A Single-Center Cohort Study From Singapore

**DOI:** 10.3389/fped.2021.801955

**Published:** 2022-01-31

**Authors:** Guan Lin Goh, Charis Shu En Lim, Rehena Sultana, Rowena De La Puerta, Victor Samuel Rajadurai, Kee Thai Yeo

**Affiliations:** ^1^Department of Neonatology, KK Women's and Children's Hospital, Singapore, Singapore; ^2^KK Research Centre, KK Women's and Children's Hospital, Singapore, Singapore; ^3^Centre for Quantitative Medicine, Duke-NUS Medical School, Singapore, Singapore; ^4^Duke-NUS Medical School, Singapore, Singapore; ^5^Yong Loo Lin School of Medicine, National University of Singapore, Singapore, Singapore; ^6^Lee Kong Chian School of Medicine, Singapore, Singapore

**Keywords:** neonatal sepsis, premature infant, mortality, risk factors, multi-organ dysfunction

## Abstract

**Objective:**

To determine the risk factors for mortality associated with late onset sepsis (LOS) among preterm very-low-birthweight (VLBW) infants.

**Study Design:**

We performed a retrospective cohort study of infants born <32 weeks gestation and <1,500 gm admitted to a Singaporean tertiary-level neonatal intensive care unit. We determined the clinical, microbial, and laboratory risk factors associated with mortality due to culture-positive LOS in this cohort.

**Results:**

A total of 1,740 infants were admitted, of which 169 (9.7%) developed LOS and 27 (16%) died. Compared to survivors, those who died had lower birth gestational age (median 24 vs. 25 weeks, *p* = 0.02) and earlier LOS occurrence (median 10 vs. 17 days, *p* = 0.007). There was no difference in the incidence of meningitis (11.1 vs. 16.9%, *p* = 0.3), NEC (18.5 vs. 14.8%, *p* = 0.6), or intestinal surgery (18.5 vs. 23.3%, *p* = 0.6) among infants who died compared to survivors. Gram-negative bacteria accounted for 21/27 (77.8%) LOS-associated deaths and almost all (13/14, 93%) fulminant episodes. The presence of multiorgan failure, as evidenced by the need for mechanical ventilation (100 vs. 79.0%, *p* = 0.008), elevated lactate (12.4 vs. 2.1 mmol/L, *p* < 0.001), and inotropic support (92.6 vs. 37.5%, *p* < 0.001), was significantly associated with mortality. Infants who died had significantly lower white blood cell (WBC) counts (median 4.2 × 10^9^/L vs. 9.9 × 10^9^/L, *p* = 0.001), lower platelet count (median 40 × 10^9^/L vs. 62 × 10^9^/L, *p* = 0.01), and higher immature to total neutrophil (I: T) ratio (0.2 vs. 0.1, *p* = 0.002). Inotrope requirement [AOR 22.4 (95%CI 2.9, 103.7)], WBC <4 × 10^9^/L [AOR 4.7 (1.7, 13.2)], and I: T ratio >0.3 [AOR 3.6 (1.3, 9.7)] were independently associated with LOS mortality.

**Conclusions:**

In a setting with predominantly Gram-negative bacterial infections, the need for inotropic support, leukopenia, and elevated I: T ratio were significantly associated with LOS mortality among preterm VLBW infants.

## Introduction

Even as neonatal intensive care continues to improve, sepsis remains a significant cause of mortality and morbidity among preterm infants (1, 2). Based on published reports over the past decade, ~12–28% of VLBW infants developed LOS (3–5) and up to 18% of those infected died (1). Recent data on LOS incidences have revealed inconsistent incidence trends in different settings—with some reporting a decline (6, 7), where others have shown a stable to increased incidence (8–12).

Gram-positive bacteria, especially coagulase-negative staphylococci, are the prevalent group of pathogens reported as the cause of LOS in many neonatal intensive care units (NICUs) (1, 3, 13–16). Even so, there have been multiple reports of units with predominant, and increasing incidence of Gram-negative LOS infections among VLBW infants from different geographical settings (4, 10, 17–21). This difference in microbial distribution is particularly important as Gram-negative infections among these high-risk infants have been associated with increased risk of fulminant sepsis and morbidities including bronchopulmonary dysplasia and neurological impairment, compared to other microorganism subtypes (1, 9, 20, 22–24). Increasing Gram-negative neonatal bacterial infections are also particularly concerning in low- and middle-income countries due to growing concerns of increasing levels of multi-drug bacterial resistance in these settings (25).

An understanding of the risk factors associated with mortality among VLBW infants with LOS is important in guiding the formulation of strategies for the treatment and prognostication of outcomes due to LOS. As such, this current study aims to determine the clinical, microbial, and laboratory risk factors associated with mortality due to LOS among VLBW infants.

## Methods

### Study Design, Setting, and Participants

We performed a retrospective cohort study of VLBW (<1,500 g) infants born <32 completed weeks gestational age and admitted to the Neonatal Intensive Care Unit (NICU) at KK Women's and Children's Hospital (KKH), Singapore, over an 11-year period (Jan 1, 2006–Dec 31, 2016). Infants with major congenital anomalies, stillbirths, and labor-room deaths were excluded. KKH is an 830-bed referral hospital and is the largest tertiary-level perinatal center in Singapore, with 40 NICU and 50 special care nursery beds. KKH provides care for ~11,500 pregnant women and 1,200 preterm infants annually.

### Data Sources

The KKH VLBW clinical database records maternal, perinatal, and neonatal information using a standardized data collection form for all live-born infants <1,500 g in the hospital. The data are cross-checked for completeness by an audit officer before submission into an electronic database. We identified all VLBW infants with positive blood and cerebrospinal (CSF) culture results from our hospital microbiology database and performed a data linkage with our VLBW database using unique national identification numbers (allocated to every baby born in Singapore).

### Variables and Definitions

LOS was defined as clinical episodes with ≥1 positive blood and/or CSF culture obtained >72 h of life, in the presence of signs or symptoms suggestive of infection. The inclusion of positive CSF cultures in the definition is based on reports where 38% of infants with pathogens isolated from CSF had negative blood cultures (13, 26). Positive cultures with *coagulase-negative staphylococci, Micrococcus, Bacillus, Corynebacterium*, and *Propionibacterium* species were considered contaminants unless ≥2 cultures were positive for the organism and/or the infant showed signs of sepsis and received intravenous antibiotics for ≥5 days. The decision to obtain blood cultures and to commence antibiotics was at the discretion of the attending physician. A minimum of 1 ml of peripheral blood was routinely obtained using an aseptic technique from the infant for blood cultures. A separate LOS episode was considered if the infant developed signs of sepsis with a positive blood culture after completion of >10-days of appropriate antibiotics. Meningitis was diagnosed if there were CNS-related symptoms and either a positive CSF culture or increased CSF white cell count (>20 × 10^6^/μL) with a positive blood culture. Mortality was attributed to LOS if it was designated as the primary cause of death by the attending physician. Fulminant sepsis was defined as death due to sepsis that occurred <72 h from antibiotics commencement (27). The empiric antibiotic regimen for LOS in our department typically includes cloxacillin and gentamicin, with escalation to cephalosporin or carbapenem if clinically indicated.

Gestational age is defined as the best obstetric estimate of completed weeks based on obstetric history, clinical examination, and antenatal ultrasound. An infant is small for gestational age if birth weight is <10th percentile according to the Fenton growth charts (28). Histologic chorioamnionitis is defined as the presence of inflammatory cells in the chorioamniotic membrane, umbilical cord, and/or the placental disc (29). Necrotizing enterocolitis (NEC) was confirmed by diagnosis at surgery/postmortem or by radiologic diagnosis with a consistent clinical history (30). Severe intraventricular hemorrhage was defined as Stage 3 and above (31).

Data on clinical course during the LOS episode was collected over a 72-h period (1 day prior to sepsis onset up to 1 day after). In this study, tachycardia was defined as heart-rate >180/min without any external stimulus or influence from drug therapy, and considered persistent if there were >2 episodes recorded within a 4-h period (32, 33). Apnea was defined as cessation of breathing for >20 s, or >10 s in the presence of bradycardia and/or desaturation (34). Temperature instability was defined as core body temperature <36°C or >38.5°C (35). Abnormal glucose levels were noted if <2.2 mmol/L or >7.8 mmol/L (35). Inotropic drug support during LOS included the usage of common inotropic agents (dopamine, dobutamine, adrenaline, and vasopressin) and/or the provision of corticosteroids for presumed adrenal insufficiency or catecholamine-resistant shock (36). Laboratory parameters, including the lowest and highest white blood cell count (WBC), platelet count, absolute neutrophil count (ANC), and the highest immature to total neutrophil (I: T) ratio during each LOS episode, were recorded. Multi-drug resistance was defined as antimicrobial resistance to at least three antimicrobial classes.

### Statistical Analysis

Differences between categories were tested using χ^2^ or Fisher's exact test (where appropriate) and Mann-Whitney *U*-test for categorical and continuous variables, respectively. Subgroup analysis was performed for fulminant sepsis and microorganism subtype. Univariate and multivariable logistic regression analysis was performed to determine associated factors for death due to sepsis. Variables with *p*-value < 0.05 were selected for the multivariable model. Stepwise variable selection methods were used to finalize the multivariable model. Continuous variables were converted to categorical variable using Youden's J–index. Quantitative associations from logistic regression were reported as odds ratio (OR) or adjusted odds ratios (AOR) with 95% confidence intervals (95% CI). Analysis of incidence trends was performed using χ^2^ test for non-linear trend. All tests were two-sided and *p*–value < 0.05 was considered statistically significant. Statistical analyses were performed using IBM SPSS Statistics version 23.0 (IBM Corporation, New York).

## Results

### Perinatal Characteristics

**Figure 1:**
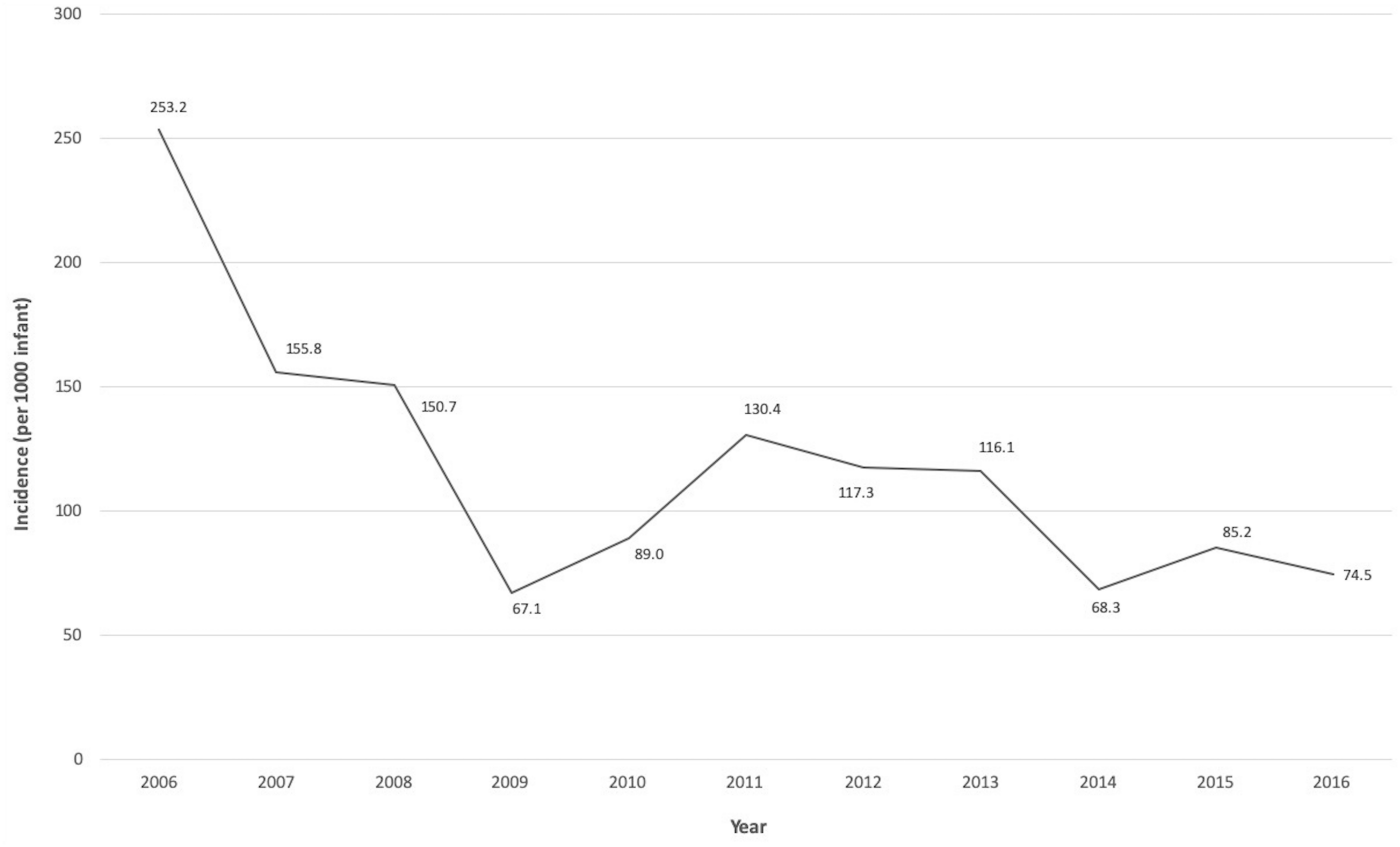
Trends of late-onset sepsis incidence over the 11-year study period.

Over the 11-year period, 1,740 VLBW infants <32 weeks gestation were admitted to the NICU. A total of 169 infants (9.7%) developed LOS, with an overall rate of 118.9 infections [standard deviation (SD) 54.5] per 1,000 infants. The LOS incidence significantly decreased over the study period from 253.2 per 1,000 infants in 2006 to 74.5 per 1,000 infants in 2016 (*p* = 0.009 for analysis of trends) (Figure 1). About half of the infants with LOS (54.4%) were born via vaginal delivery, 79.3% were <28 weeks gestation, 85.2% had birthweight ≤ 1,000 g, 59.8% were male and 94.7% had insertion of a central venous catheter. Up to 88.2% of mothers from these infants received antenatal steroids, 51.5% had histologic placental chorioamnionitis, and 46.2% received intrapartum antibiotics. A total of 32 infants (19%) had ≥2 LOS episodes and 4 infants (2.4%) had 3 episodes. Among those with multiple LOS, all were <28 weeks gestation with median birthweight of 726 g [Interquartile range (IQR) 646–810]. All infants in this study (*n* = 169) received an average of 4 separate (SD 2) exposures to antibiotics for concerns of infections prior to initial discharge or death.

**Figure 2:**
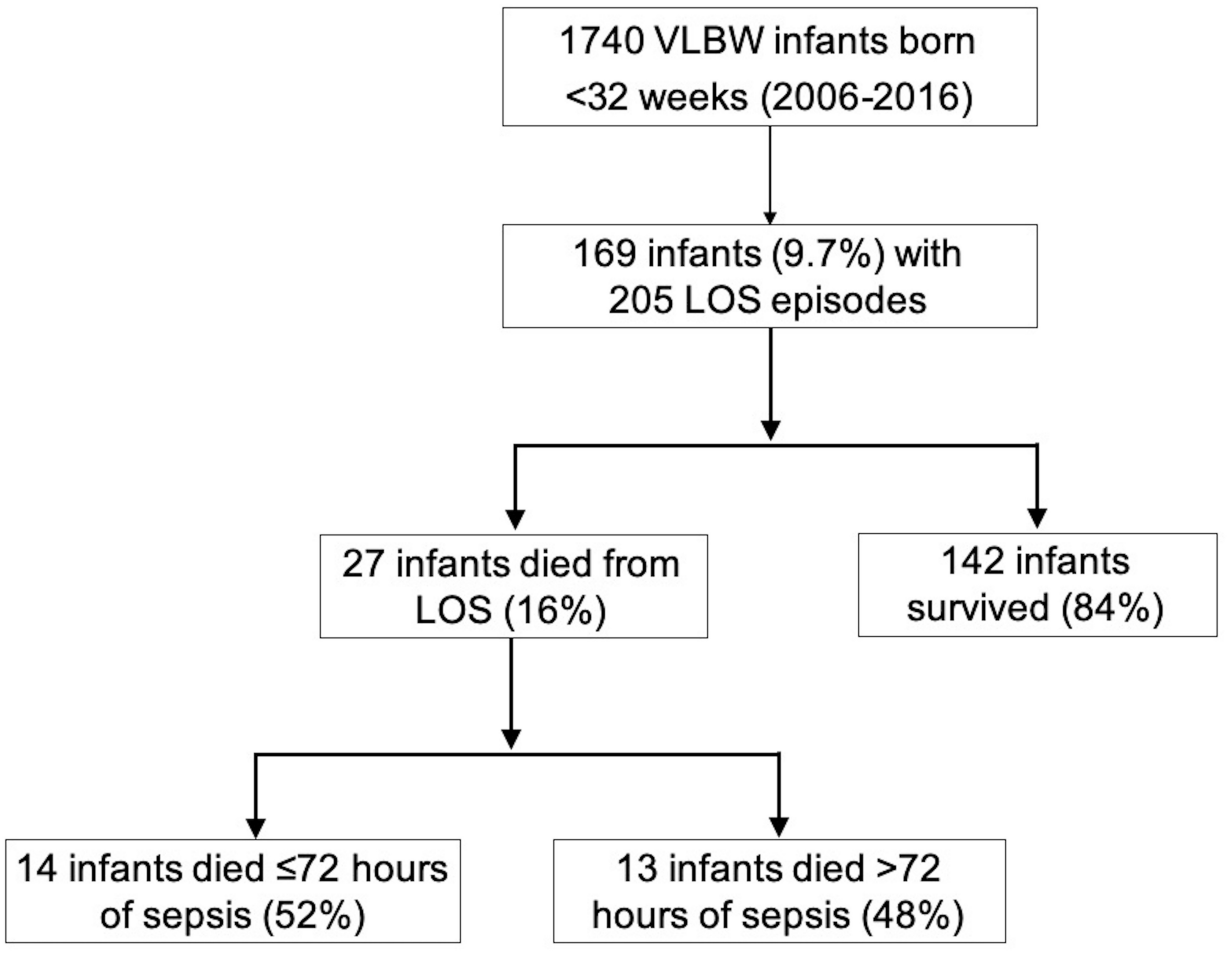
Outcomes of preterm VLBW infants with late-onset sepsis.

A total of 27 (16%) infants died due to complications of LOS (Figure 2). The proportion of LOS deaths over the total number of LOS cases over the first 5 years was not different compared to the latter 5 years [14/95 (14.7%) vs. 11/64 (17.3%), *p* = 0.8). Compared to LOS survivors, those who died were born at lower gestational age (median 24 vs. 25 weeks, *p* = 0.02) (Table 1), and had earlier LOS occurrence (median age 10 vs. 17 days, *p* = 0.007). There was no difference in the incidence of meningitis (11.1 vs. 16.9%, *p* = 0.3), NEC (18.5 vs. 14.8%, *p* = 0.6), or intestinal surgery (18.5 vs. 23.3%, *p* = 0.6) among infants who died compared to survivors (Table 1; Supplementary Table 1). Slightly more than half of the LOS deaths (14/27, 52%) were fulminant. Comparing infants with fulminant and non-fulminant sepsis, there were generally no significant differences in pregnancy complications, antenatal interventions, and neonatal characteristics (Supplementary Table 2), and no difference in bacteremia onset (median 8.5 vs. 9 days of life, respectively, *p* = 0.5). A smaller proportion of infants with fulminant sepsis were born via vaginal delivery compared to those without (50 vs. 92.3%, *p* = 0.02). Among infants who died from non-fulminant sepsis, the median duration from sepsis onset to death for this group was 5 days (range 4–18).

**Table 1:** Perinatal characteristics of preterm VLBW infants with late onset sepsis (*n* = 169).

	**Death due to sepsis** ** (*n* = 27)**	**Survivors of sepsis** ** (*n* = 142)**	***P*-value**
Median maternal age (IQR)	33 (27.36)	31 (28.35)	0.8
Antepartum hemorrhage (%)	9 (33.3)	29 (20.4)	0.1
Pregnancy-induced hypertension (%)	5 (18.5)	23 (16.2)	0.8
Prolonged rupture of membrane (%)	5 (18.5)	44 (31.0)	0.2
Antenatal steroids (%)	21 (77.8)	128 (90.1)	0.07
Histologic chorioamnionitis (%)	15 (55.6)	72 (51.1)	0.7
Maternal antibiotics (%)	11 (40.7)	67 (47.2)	0.3
Male (%)	18 (66.7)	83 (58.5)	0.4
Vaginal delivery (%)	19 (70.4)	73 (51.4)	0.07
Median birthweight (IQR)	695 (615, 786)	758 (650, 911)	0.07
Median gestational age (IQR)	24 (24, 25)	25 (24, 27)	0.02
Multiple gestation (%)	7 (25.9)	34 (23.9)	0.8
Small for gestational age (%)	5 (18.5)	26 (18.3)	1.0
5-min Apgar <7 (%)	9 (33.3)	26 (18.3)	0.07
Intestinal surgery (%)^*^	5 (18.5)	33 (23.3)	0.6

*IQR: inter-quartile range*.

* *Excludes elective inguinal hernia surgery*.

### Microorganisms

Gram-negative bacteria were the most common microorganisms isolated with each LOS episode (131/205, 64%), with similar proportions of Gram-positive and fungal organisms (37/205, 18%) (Table 2). Gram-negative infections were also more common among sepsis episodes leading to death, accounting for 77.8% (21/27) of such episodes compared to 61.8% (110/178) of episodes among survivors (*p* = 0.1). *Klebsiella* spp was the most common causative bacteria in both groups. Gram-positive infections were more common among LOS episodes with survivors compared to those resulting in death (19.7 vs. 7.4%, *p* = 0.2), with *Coagulase negative staphylococcus* being the predominant organism. *Candida* spp accounted for all the fungal infections in our population with *Candida parasilopsis* being the most common. Among fulminant LOS episodes, almost all were Gram-negative bacteria (13/14, 93%) with *Acinetobacter, Klebsiella, Pseudomonas* spp (2 infections (21.4%) each) being the most common.

**Table 2:** Distribution of microorganisms isolated in late onset sepsis episodes (*n* = 205).

	**Sepsis episodes leading to death** ** (*n* = 27)**	**Sepsis episodes with survivors** ** (*n* = 178)**	**Total** ** (*n* = 205)**
*Gram positive*	2 (7.4)	35 (19.7)	37 (18.0)
S. agalactiae	0	3 (1.5)	3 (1.4)
S. aureus	1 (3.7)	14 (7.9)	15 (7.3)
E. faecalis	0	1 (0.6)	1 (0.5)
Bacillus spp	0	1 (0.6)	1 (0.5)
CONS	1 (3.7)	16 (9.0)	17 (8.3)
*Gram negative*	21 (77.8)	110 (61.8)	131 (63.9)
Acinetobacter spp	4 (14.8)	16 (9.0)	20 (9.8)
E. coli	4 (14.8)	15 (8.4)	19 (9.3)
E. meningoseptica	2 (7.4)	4 (2.2)	6 (2.9)
Enterobacter spp	1 (3.7)	25 (14.1)	26 (12.7)
Klebsiella spp	5 (18.5)	28 (15.8)	33 (16.1)
Serratia spp	0	12 (6.7)	12 (5.9)
Pseudomonas spp	4 (14.8)	8 (4.5)	12 (5.9)
Burkholderia cepacia	0	1 (0.6)	1 (0.5)
Morganella morganii	0	1 (0.6)	1 (0.5)
Pantoea agglomerans	1 (3.7)	0	1 (0.5)
*Fungal infection*	4 (14.8)	33 (18.5)	37 (18.0)
Candida parasilopsis	1 (3.7)	27 (15.2)	28 (13.7)
Candida albicans	3 (11.1)	3 (1.7)	6 (2.9)
Candida glabrata	0	3 (1.7)	3 (1.4)

*CONS: Coagulase negative staphylococcus*.

There was no difference in the incidence of multi-drug resistance in infecting microorganism between infants who died compared to survivors of LOS (26 vs. 17%, *p* = 0.2). For the six most common groups of Gram-negative bacteria isolated (*Acinetobacter* spp, *E. coli, Enterobacter* spp, *Klebsiella* spp, *Serratia* spp, *Pseudomonas* spp), there was no significant difference in the incidence of gentamicin resistance (47.6 vs. 38.9%, *p* = 0.5) and cefotaxime/ceftriaxone resistance (61.8 vs. 44.4%, *p* = 0.2) between infants who died compared to survivors, respectively. Only 1 out of 37 (2.7%) fungal LOS episode had prior exposure to cephalosporins therapy, compared to 45 out of 168 (26.8%) bacterial LOS episodes.

### Clinical Course During LOS Episodes

Compared to those with survivors, episodes resulting in death were more likely to be complicated by seizures (59.3 vs. 6.3%, *p* ≤ 0.001), require mechanical ventilation (100 vs. 79%, *p* = 0.008), require inotropic support (92.6 vs. 37.5%, *p* ≤ 0.001), and have higher median lactate values (12.4 vs. 2.1 mmol/L, *p* < 0.001) (Table 3). The median number of inotropic agents required was also significantly higher in episodes leading to death compared to those with survivors (2 vs. 0, *p* ≤ 0.001).

**Table 3:** Clinical course during late onset sepsis episodes stratified by those with survival and those resulting in death (*n* = 205).

	**Episodes resulting in death** ** (*n* = 27)**	**Episodes with survival** ** (*n* = 178)**	***P*-value**
Median number of apneic episodes (IQR)	1 (0,3)	1 (0,3)	0.9
Persistent tachycardia (heart rate >180) (%)	17 (89.5)	73 (79.3)	0.3
Temperature instability (T <36°C or T >38.5°C) (%)	2 (7.4)	7 (4.0)	0.4
Abnormal glucose (<2.2 to >7.8 mmol/L) (%)	24 (96.2)	146 (83.4)	0.09
Lethargy/irritability (%)	4 (14.8)	22 (12.5)	0.7
Seizures (%)	16 (59.3)	11 (6.3)	<0.001
Required mechanical ventilation (%)	27 (100)	139 (79.0)	0.008
Inotropic support (%)	25 (92.6)	66 (37.5)	<0.001
Median number of inotropic agents (IQR)	2 (2, 4)	0 (0, 1)	<0.001
Median highest lactate value, mmol/L (IQR)	12.4 (4.6, 15.9)	2.1 (1.6, 3.4)	<0.001
pH <7.25 (%)	25 (96.2)	128 (72.7)	0.009
MDRO (%)	7 (25.9)	30 (16.9)	0.3

*MDRO, multi-drug resistant organism; IQR, interquartile range*.

### Laboratory Findings

**Figure 3:**
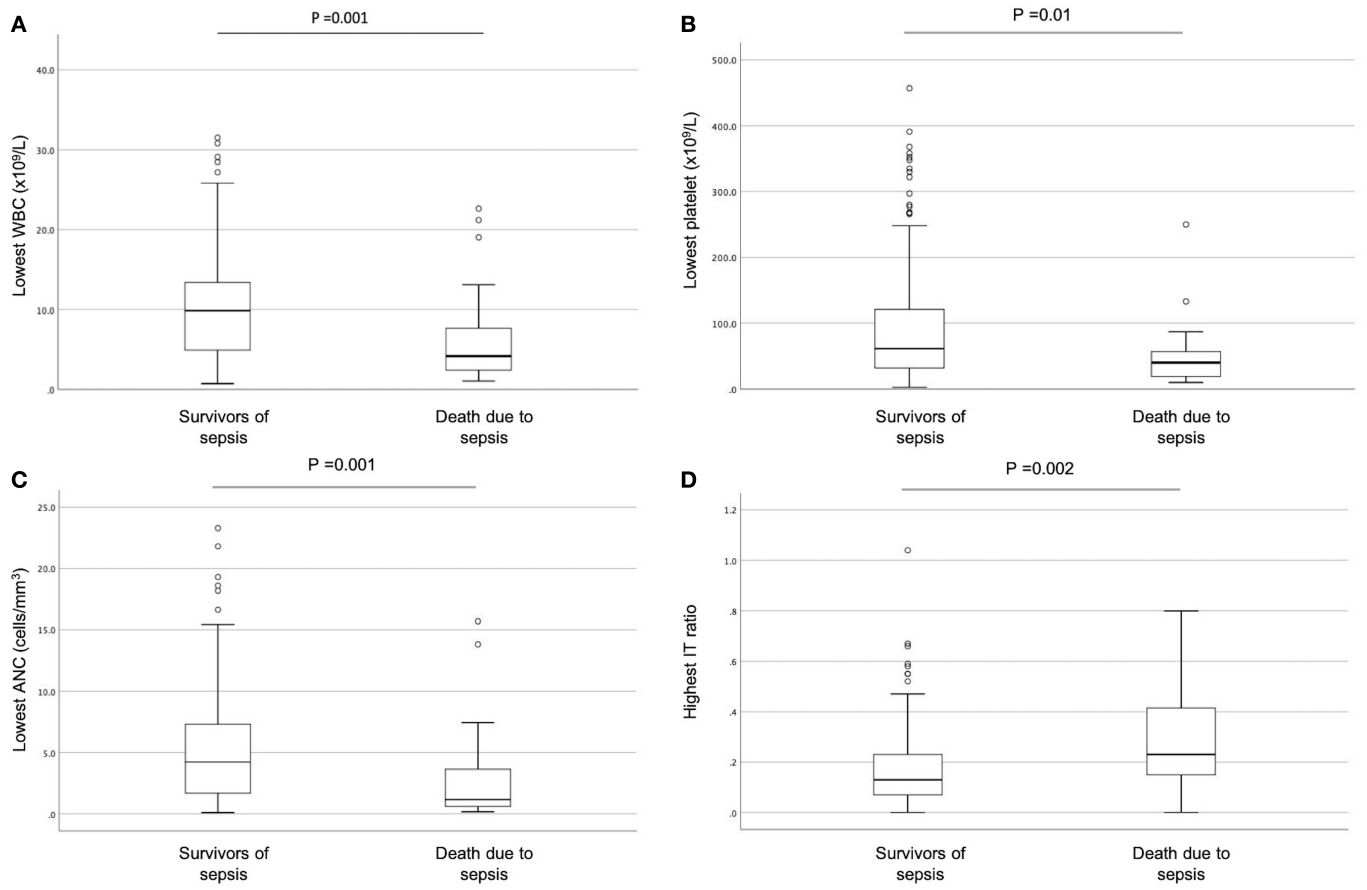
Comparisons of laboratory parameters between VLBW infants who died and infants who survived late onset sepsis. Comparisons of the following parameters during LOS episode: **(A)** lowest WBC, **(B)** lowest platelets, **(C)** lowest absolute neutrophil count, **(D)** highest I: T ratio. The open circles in the graphs indicate outliers.

Compared to survivors, infants who died had significantly lower WBC [median 4.2 × 10^9^/L (IQR 2.4–7.7) vs. 9.9 × 10^9^/L (IQR 4.9–13.4), *p* = 0.001), lower platelet count [median 40 × 10^9^/L (IQR 19–57) vs. 62 × 10^9^/L (32–121), *p* = 0.01], lower ANC [median 1.2 cells/mm^3^ (IQR 0.6–3.6) vs. 4.2 cells/mm^3^ (IQR 1.7–7.3), *p* = 0.001], and higher I:T ratio [median 0.2 (IQR 0.2–0.4) vs. 0.1 (IQR 0.1–0.2), *p* = 0.002] (Figure 3). Infants with Gram-negative and invasive fungal infections were also found to have lower white cell count, lower platelet count, lower absolute neutrophil count, and higher I:T ratio compared to those with Gram-positive bacteremia (Supplementary Figure 1).

### Predictors of Death due to LOS

Using multivariable logistic regression analysis, we determined that inotrope requirement [AOR 22.4 (2.9, 103.7)], WBC <4 × 10^9^/L [AOR 4.7 (1.7, 13.2)] and I: T ratio ≥0.3 [AOR 3.6 (1.3, 9.7)] were significantly associated with mortality from LOS in VLBW infants (Table 4).

**Table 4:** Multivariable logistic regression model of clinical, microbiologic, and laboratory parameters associated with death due to LOS.

**Variables**	**AOR (95% CI)**	***P*-value**
Inotropic support	22.4 (2.9.103.7)	<0.0001
WBC count <4 ×10^9^/L	4.7 (1.7.13.2)	0.003
I:T ratio ≥0.3	3.6 (1.3.9.7)	0.01

*WBC, white blood cell; I: T ratio, immature to total neutrophil ratio*.

## Discussion

Concurrent with trends of improved survival of VLBW infants over the past few decades (37) there have been varying reports on LOS incidence trends in this group of infants (6–12). Our decreasing LOS trends and the overall incidence of 118.9 per 1,000 infants over 11 years is comparable to other recent reports, with reported LOS incidence ranging from 72 to 339 episodes per 1,000 admissions (3–5, 20, 22). Similarly, the LOS mortality rate of 16% seen in our study is within the range seen in other reports, from 9 to 21% (1–4, 18, 38). Importantly, the proportion of LOS due to Gram-negative bacteria in our study (64%) is significantly higher than that in other reports (16–48%) (20, 24).

Available reports on LOS from individual neonatal units and neonatal networks from the US, Europe, Australia, and New Zealand (1–4, 6, 14, 16, 39) have reported a predominance of Gram-positive bacteria as a causal LOS microorganism among VLBW infants, with rates ranging from 55 to 84%. These data are in sharp contrast to our study, where Gram-negative bacteria is seen in up to 64% of LOS episodes and is associated with 78% of LOS mortality. Importantly, almost all (13/14, 93%) who had fulminant LOS were infected by a Gram-negative organism. The predominance of Gram-negative organisms in LOS have been reported in other units, many from NICUs in low- and middle-income countries (5, 18, 25, 40). Shah et al. hypothesized that the increasing usage of antibiotics in the antepartum and neonatal period may have contributed to the increasing trend of Gram-negative infections seen in these units (10). The antibiotic exposure during early life could lead to altered neonatal mucosal colonization and a predominance of Gram-negative bacteria, resulting in an increased risk of Gram-negative infections (41, 42). Also, prolonged and inappropriate antibiotic usage could lead to emergence of multidrug resistance Gram-negative bacteria which have been associated with increased risk of infectious complications and death (43). In our study, there was a relatively high exposure to antibiotics among our preterm infants, where almost half of our infants (46.1%) had exposure to intrapartum antibiotics and each infant received an average of four courses of antibiotics during their admission. However, we did not find significant difference in the rates of multi-drug resistance between infants who died compared to survivors of sepsis.

Previous studies have associated Gram-negative LOS infections with increased risk of fulminant sepsis (1, 24). In a retrospective 10-year single-center study from the US, Karlowicz et al. reported that Gram-negative organisms accounted for 69% of fulminant sepsis cases with *Pseudomonas* spp accounting for up to 56% of fulminant LOS episodes (24). These findings are consistent with our study which found that *Pseudomonas, Klebsiella*, and *Acinetobacter* spp accounted for up to 64% of all fulminant LOS episodes. The independent effect of infecting Gram-negative organisms on early LOS mortality was previously reported, with *Pseudomonas, Klebsiella*, and *Serratia* being associated with the highest risk of early mortality (44). The differential risks for fulminant mortality may be related to the potential of selected organism types to form biofilms, exhibit multi-drug resistance, and demonstrate hypervirulence (45).

Studies have reported an invasive fungal infection incidence of 2.4–18%, and a mortality rate of 26–33% (1, 3–5, 14, 23, 38). The incidence of fungal sepsis in this study was comparable but we reported a lower mortality rate. The incidence of fungal infection in our cohort is likely modulated by the introduction of fluconazole prophylaxis in our center in 2009. Most episodes of fungal sepsis occurred prior to this change, with only 3 episodes occurring after the start of fungal prophylaxis of all infants <800 g and/or those with invasive intravenous central catheter. This observation is consistent with current evidence indicating the efficacy of antifungal prophylaxis in reducing invasive candidiasis in preterm infants.

Infants who died from LOS were of significantly lower gestational age and had earlier occurrence of sepsis. It is well-established that infants of lower gestational age are more likely to require mechanical ventilation (46), longer duration of central venous catheter (15), increased antimicrobial exposure (47), and longer duration of hospitalization, which renders them more susceptible to nosocomial infections. Even so, gestational age or timing of LOS were not independently associated with death due to sepsis in our analysis. We determined that the need for inotropes, significant neutropenia, and an elevated I: T ratio were all independently associated with death due to LOS. Progression toward multi-organ injury and dysfunction have been recently evaluated to predict LOS mortality among preterm, VLBW infants (27, 36, 48). We similarly found that LOS episodes resulting in death were associated with increased need for mechanical ventilation and inotropic support. Additionally, we noted a higher likelihood of neurological dysfunction with seizures and early evidence of metabolic dysfunction with elevated lactate and significant acidosis among those who died. In our analysis, the need for inotropic support was the most important factor associated with the risk of death among preterm VLBW infants with LOS, consistent with findings in other studies (36, 48).

Compared to survivors of LOS in our study, infants who died had significantly lower white cell counts, lower absolute neutrophil counts, lower platelet counts, and higher I:T ratio. These changes are attributed to increased neutrophil demargination during infection combined with increased cellular destruction in the peripheral circulation and bone marrow depression, resulting in reduced numbers of mature neutrophils (38). The preterm infant's reduced ability to accelerate neutrophil and thrombocyte production during infection likely compounds the derangement in the counts (23, 49–51). The low G-CSF levels seen in neonatal sepsis also likely contributes to these findings (51). Consistent with our results, previous studies have also reported that infants had higher incidence of neutropenia (52), higher I:T ratio (53), and lower platelet count (49), especially in Gram-negative infections. These derangements in laboratory parameters are potentially important in the clinical setting as an indicator of increased risk of disease severity and mortality in LOS.

Our study was limited by the small sample size and the single center design. Even so, our cohort involving >200 LOS episodes analyzed over an 11-year period provided a reasonable number from which detailed perinatal, clinical data from the LOS episodes could be obtained and important inferences can be made. However, the lower numbers of LOS bacteremia precluded us from undertaking further analysis to determine differential risk of infection and mortality between different types and subgroups of bacteria. Other limitations include the lack of data on breastmilk intake rates, which is known to reduce the risk of sepsis and allow for the development of the appropriate intestinal flora and microbial predominance among preterm infants.

## Conclusion

In our setting with a predominance of Gram-negative bacterial LOS among preterm VLBW infants, having any requirement for inotropic support, low WBC <4 × 10^9^/L and an I: T ratio ≥0.3 are significantly associated with increased risk of death. Our data also suggests that empiric antimicrobial coverage for LOS in similar settings needs to treat Gram-negative organisms broadly and effectively.

## Data Availability Statement

The original contributions presented in the study are included in the article/Supplementary Material, further inquiries can be directed to the corresponding author/s.

## Ethics Statement

The studies involving human participants were reviewed and approved by SingHealth Centralized Institutional Review Board (CIRB No. 2017/2761). Written informed consent from the participants' legal guardian/next of kin was not required to participate in this study in accordance with the national legislation and the institutional requirements.

## Author Contributions

GG and KY conceptualized and designed the study, coordinated and acquired the data, carried out the initial analyses, drafted the initial manuscript, and critically revised the manuscript. CL and RD coordinated and contributed to the acquisition of data, and critically revised the manuscript. RS was responsible for the statistical analysis, data interpretation, and critical revision of the manuscript. VR contributed to the analysis, interpretation of data, and critical revision of the manuscript. All authors approved the final manuscript as submitted and agree to be accountable for all aspects of the work.

## Conflict of Interest

The authors declare that the research was conducted in the absence of any commercial or financial relationships that could be construed as a potential conflict of interest.

## Publisher's Note

All claims expressed in this article are solely those of the authors and do not necessarily represent those of their affiliated organizations, or those of the publisher, the editors and the reviewers. Any product that may be evaluated in this article, or claim that may be made by its manufacturer, is not guaranteed or endorsed by the publisher.

## Supplementary Material

Supplementary Table 1 Selected major neonatal morbidities of VLBW infants with late onset sepsis.

Supplementary Table 2 Comparison of perinatal characteristics of VLBW infants with fulminant and non-fulminant late onset sepsis (*n* = 27).

Supplementary Figure 1 Comparisons of laboratory parameters associated with different infecting microorganism subtypes. Comparisons of the following parameters during LOS episode: **(A)** lowest WBC, **(B)** lowest platelets, **(C)** lowest absolute neutrophil count, **(D)** highest I: T ratio.
