# Supplementary material for: Risk Factors for Mortality From Late-Onset Sepsis Among Preterm Very-Low-Birthweight Infants: A Single-Center Cohort Study From Singapore
Source: Front Pediatr. 2022 Jan 31;9:801955. doi: 10.3389/fped.2021.801955 (PMC8841856; doi:10.3389/fped.2021.801955)
Supplement: Supplementary Table 1 — Selected major neonatal morbidities of VLBW infants with late onset sepsis. [file Data_Sheet_1.docx]

**Supplemental Table 1. Selected major neonatal morbidities of VLBW infants with late onset sepsis**

|  | **Infant with death due to sepsis (n=27)** | **Survivors of sepsis (n=142)** | **p-value** |
| --- | --- | --- | --- |
| Meningitis (%) | 3 (11.1) | 24 (16.9) | 0.3 |
| Necrotizing enterocolitis (%) | 5 (18.5) | 21 (14.8) | 0.6 |
| Severe intraventricular haemorrhage (%) | 13 (48.1) | 16 (11.3) | <0.001 |
| Bronchopulmonary dysplasia  O2 dependency at 28 day of life (%)  O2 dependency at corrected 36 weeks’ gestation (%) | 5* (18.5)  1^ (3.7) | 120 (84.5)  91 (64.1) | <0.001  <0.001 |

*20 infants (74.1%) died prior to 28 days of life

^23 infants (85.2%) died prior to 36 weeks’ corrected gestation

**Table 2. Comparison of perinatal characteristics of VLBW infants with fulminant and non-fulminant late onset sepsis (n=27)**

|  | **Infants with fulminant sepsis (n=14)** | **Infants with non-fulminant sepsis (n=13)** | **p value** |
| --- | --- | --- | --- |
| Median maternal age (range) | 33 (17,45) | 31 (18,40) | 0.5 |
| Antepartum haemorrhage (%) | 7 (50.0) | 2 (15.4) | 0.06 |
| Pregnancy-induced hypertension (%) | 4 (28.6) | 1 (7.7) | 0.2 |
| Prolonged rupture of membrane (%) | 4 (28.6) | 1 (7.7) | 0.1 |
| Antenatal steroids (%) | 12 (85.7) | 9 (69.2) | 0.3 |
| Histologic chorioamnionitis (%) | 7 (50.0) | 8 (61.5) | 0.5 |
| Maternal antibiotics (%) | 7 (50.0) | 4 (30.8) | 0.3 |
| Male (%) | 9 (64.3) | 9 (69.2) | 0.8 |
| Vaginal delivery (%) | 7 (50.0) | 12 (92.3) | 0.02 |
| Median birthweight (range) | 681 (450,970) | 714 (592,1070) | 0.2 |
| Gestational age <28 weeks (%) | 13 (92.9) | 13 (100) | 0.3 |
| Median gestational age (range) | 24 (23,29) | 25 (24,26) | 0.3 |
| Multiple gestation (%) | 4 (28.6) | 3 (23.1) | 0.7 |
| Small for gestational age (%) | 4 (28.6) | 1 (7.7) | 0.1 |
| 5-min Apgar <7 (%) | 5 (35.7) | 4 (30.8) | 0.8 |
| Meningitis (%) | 2 (14.3) | 5 (38.5) | 0.2 |
| Presence of central venous catheter (%) | 8 (57.1) | 10 (76.9) | 0.2 |
