# Supplementary figures and images for: Risk Factors for Mortality From Late-Onset Sepsis Among Preterm Very-Low-Birthweight Infants: A Single-Center Cohort Study From Singapore
Source: Front Pediatr. 2022 Jan 31;9:801955. doi: 10.3389/fped.2021.801955 (PMC8841856; doi:10.3389/fped.2021.801955)

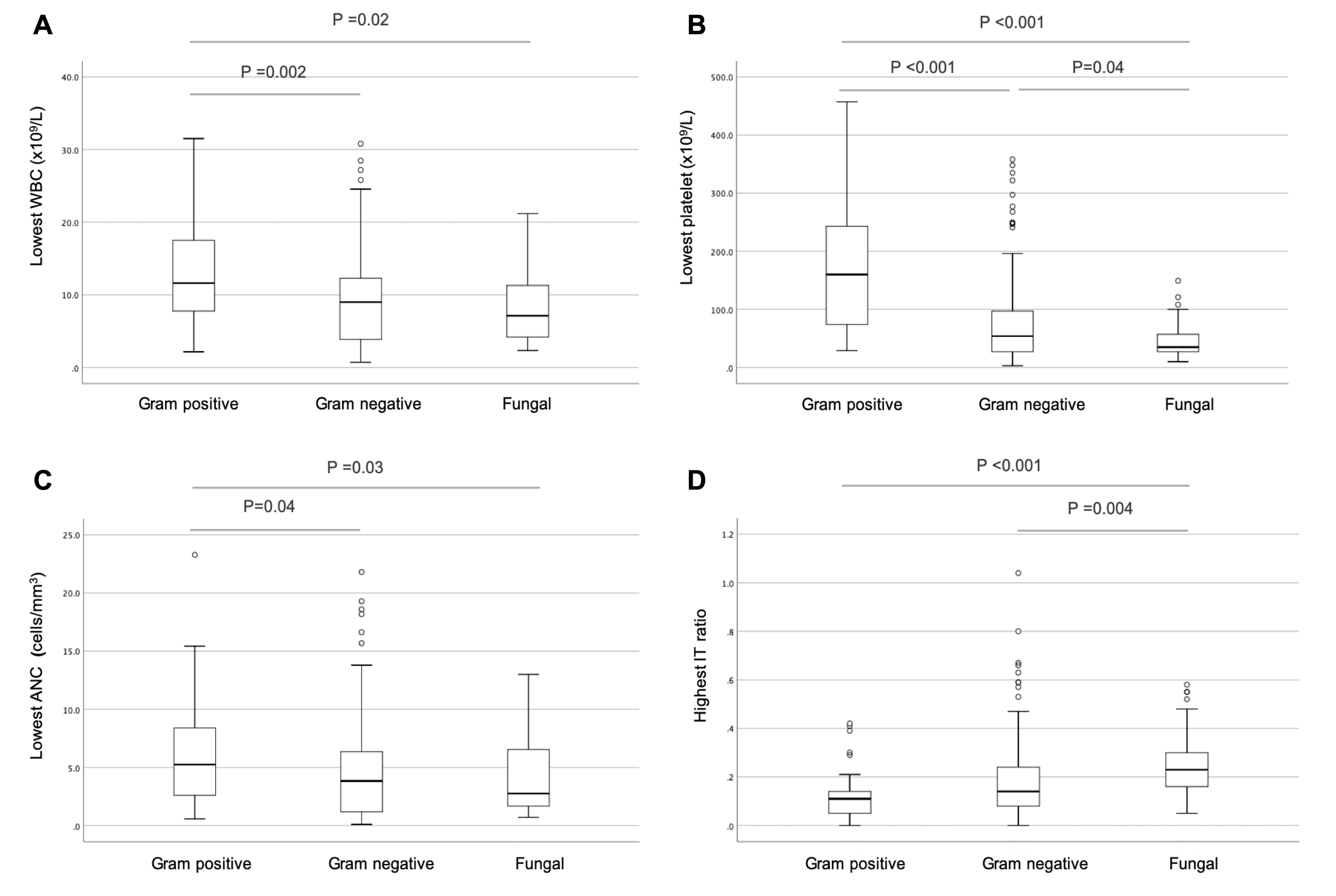

Supplement: Supplementary Figure 1 — Comparisons of laboratory parameters associated with different infecting microorganism subtypes. Comparisons of the following parameters during LOS episode: (A) lowest WBC, (B) lowest platelets, (C) lowest absolute neutrophil count, (D) highest I: T ratio. [file Image_1.JPEG]
